# Notes and Comments

**Published:** 1922-06

**Authors:** 


THE
### HOSPITAL and HEALTH
REVIEW

### ANTI-EPIDEMIC WORK IN EASTERN EUROPE

APART from the National Public Health Services concerned, the chief agencies engaged in anti-epidemic work in Poland and Eastern Europe are :—

(*a*) The Epidemic Commission of the League of Nations.

(*b*) Numerous voluntary organisations, such as the International Union for Helping the Children, the Italian, French, Swedish and International Red Cross Societies, the Save the Children Fund, &c.

(*c*) The Health Section of the League of Nations, which has earmarked 25,000 gold francs of its 1922 estimates for anti-typhus work in Poland.

So far as Poland is concerned, says a correspondent, an attempt has been made to co-ordinate all this official and voluntary effort by the establishment of an Advisory Board, which held its first meeting in Warsaw in April, 1921. The Board contains representatives of the Epidemics Commission, the social section of the Secretariat of the League of Nations, the International Committee of the Red Cross Society, the League of Red Cross Societies, and the Office International d'Hygiène Publique, and its meetings are attended by representatives of the Polish Ministry of Health.

Attention was drawn, early in 1919, to the serious prevalence of typhus and other epidemic diseases in Eastern Europe, and to the danger of their spread westward. A small Committee of Inquiry was sent to Poland by the League of Red Cross Societies to survey the position and to make recommendations. This Committee confirmed the reports as to the gravity of the situation and emphasised the need for action on a much more comprehensive scale than was possible with local and voluntary effort. The question was considered later at a meeting of the Council of the League of Nations in Paris in March, 1920, and a resolution adopted recognising the need for immediate intervention in the interests not only of Poland but of Europe generally, and inviting the International Health Conference to submit proposals with a view to concerted action. The proposals ultimately submitted involved the appointment of an Epidemics Commission, and an expenditure of £3,250,000. (For immediate purposes £2,000,000 was suggested as a minimum.)

At the meeting of the Supreme Council at Rome in May, 1920, the appointment of the Epidemics Commission was agreed to, but the main proposals, although viewed with favour, were apparently thought to be too ambitious for official adoption. Instead, an unofficial appeal was made for voluntary contributions, and Great Britain promised £50,000 on condition that four other countries subscribed similar amounts. The response was unsatisfactory, and in spite of subsequent appeals nothing happened till December, 1920, when, in view of the gravity of the Polish situation, Great Britain waived her condition and subscribed £50,000 forthwith. A few other countries followed suit, and the funds thus placed at the disposal of the Epidemics Commission in time amounted to £126,397. In addition the League of Red Cross Societies contributed £10,000 together with large quantities of clothing and certain hospital requirements. The activities of the Commission have been confined mainly to the Grodno-Lida-Wolkowysk-Brest Litovsk-Bialystok area in N.E. Poland. In addition special help has been given to the Polish Health Administration in securing the efficient working of the two large and important quarantine stations at Rowno and Baranowicze. Through these most of the returning Bolshevik and Polish prisoners of war pass. There is close co-operation with the Polish Health Authority, and the latter gives the Commission every facility for inspection. Up to August 1, 1921, the Commission spent £119,067, mainly on hospitals, supplies and equipment, motor ambulances and other vehicles, clothing, drugs, soap, and bathing and disinfecting establishments. Owing to exhaustion of funds the work of the Commission is now seriously hampered.

The heavy strain placed upon the Polish sanitary cordon will be appreciated when we consider the enormous mass of infection across the border in Russia. It is estimated that during the years 1919 and 1920 some 20,000,000 cases of typhus occurred in the territories of the ancient Russian Empire (excluding Poland, Lithuania, Finland and Latvia). Only a small proportion of these were notified. The annual pre-war average was about 150,000. There was a decline in 1921, but recent reports are ominous. Relapsing fever has followed the lines of development of typhus. Cholera, for some unexplained reason, came to an end suddenly in June, 1921, in the middle of what is usually the epidemic season. One important factor leading to the localisation of epidemics in Russia is the difficult transport situation.

The main problem of the refugee in Russia is that of the peasant of Western origin. Large numbers are still waiting to be repatriated. A conservative estimate is 1,500,000. During the last few months, owing to the breakdown of the Russian evacuation system (the "Certfrevac"), the Polish quarantine stations at Rowno and Baranowicze have proved unable to cope with the influx of refugees, so that cases of typhus have evaded the vigilance of the frontier.

The Conference at Warsaw this spring drew up a plan of campaign, estimated to cost £1,500,000, for definitely overcoming the menace of epidemics. This plan is divided into (*a*) immediate measures for checking the spread of epidemics beyond the borders of Russia, and (*b*) a scheme of aid to the Russia and Ukrainian health services in an attack on the centres of infection. In this connection the Conference registered its opinion that a purely defensive campaign on the frontiers of Russia would merely prolong the problem indefinitely, thus causing more expenditure in the end than a decisive anti-epidemic offensive undertaken for the purpose of quelling the epidemics at their source. The payment of the necessary monies for this campaign should be on a scale similar to that for payment of the League budget by members of the League. The Conference also resolved that the execution of these measures and the control of the campaign should be entrusted to the League Health Organisation and Epidemics Commission, and added a rider insisting on the necessity for all States taking part being represented on these organisations. The Soviet Russian and Ukrainian delegations alone abstained from voting on this measure, and presented, instead, an amendment asking that a special international commission, on which all the Powers concerned should be equally represented, be entrusted with control. Finally, the Conference approved a draft designed to serve as a model for a series of sanitary conventions to be concluded between the Central and East European States taking an active part in the work. Such a convention is already being negotiated between Soviet Russia and Poland, and at the Conference the delegations of the Baltic States, Finland, Czecho-Slovakia, Germany, Poland, Roumania and Russia declared their readiness to begin mutual negotiations immediately, with a view to concluding a series of similar sanitary conventions. The Conference unanimously approved a resolution moved by the Czecho-Slovak delegation, declaring that if disputes should arise between contracting States on points concerning these conventions, the Health Section of the League should be constituted as mediator, without prejudice, however, to the right of any State to have recourse to a different procedure.

---

### WELLINGTON HOUSE, 1911-1922.

THE removal last month of the headquarter officials of the Insurance Department from Wellington House to the Ministry of Health means the end of the first chapter of the history of national insurance. At Wellington House have worked probably more distinguished civil servants since 1911 than in any other Government Department. Many of them are well known to the public, for after being associated with national insurance in its early days they have made their names in other administrative appointments. The late Sir Robert Morant was the first Chairman of the Commission, and he was largely responsible for the selection of the staff. With him were Sir Warren Fisher, now Permanent Secretary of the Treasury; Sir John Bradbury, now head of the Reparations Commission, and the signatory of the original pound notes; Sir John Anderson, recently Under-Secretary for Ireland and now Under-Secretary at the Colonial Office; Sir Claude Schuster, now secretary to the Lord Chancellor; Sir David Shackleton, now Labour Adviser to the Ministry of Labour; Mr. S. P. Vivian, now Registrar-General; Mr. E. A. Gowers, now Secretary of the Mines Department; Mr. R. A. Johnson, now Deputy-Master of the Mint; Mr. R. E. Harwood, now Deputy Treasurer to the King; Sir William Sutherland, now Chancellor of the

Duchy of Lancaster; Mr. L. G. Brock, now an Assistant Secretary at the Ministry of Health and Secretary of the Voluntary Hospitals Commission; Sir H. Noel Bunbury, now Accountant-General of the Post Office; Mr. J. A. Salter, now with the Reparations Commission; Mr. J. Rae, now Assistant Secretary of the Treasury; and Sir Alfred Woodgate—all took their share in the creation of national insurance. Mr. Lloyd George worked at Wellington House when he was introducing the Bill. Mr. C. F. G. Masterman, Capt. Wedgwood Benn, Mr. Charles Roberts and Sir Edwin Cornwall are among the politicians who occupied that building. It is no longer to be in Government service, but will always be associated with the opening phase of national insurance administration.

### THE SCHOOL OF HYGIENE.

PRACTICALLY all the daily papers have fallen into the mistake of calling the proposed School of Hygiene, to be built on a central site bounded by Gower Street, Keppel Street and Market Street, Bloomsbury, "the London School." Although the buildings will be in London, the work carried on will be international in its scope. The trustees of the Rockefeller Foundation have generously given the sum of two million dollars, with a view to forwarding the interests of public health both at home and abroad, and the British Government has accepted the responsibility for providing for the staffing and maintenance of the school at a cost of not less than £25,000 for the next five years, because of its international importance.

This is the main object kept in view by the committee that has been appointed, of which Sir Arthur Robinson, Permanent Secretary of the Ministry of Health, is chairman. The other members are: Sir Frank Baines, who brings special experience of public works to bear upon the building problem; Sir Walter Fletcher, of the Medical Research Council; Sir William Leishman, at one time President of the Society of Tropical Medicine and Hygiene; Sir George Newman, Chief Medical Officer of the Ministry of Health; Sir Cooper Perry, who has intimate knowledge of the needs of the University of London; Sir Herbert J. Read, one of the British plenipotentiaries at the Sleeping Sickness Conference; Dr. H. H. Dale, head of the Department of Biochemistry and Pharmacology; and the secretary, Dr. H. Meredith Richards, of the Ministry of Health.

The records of the members of the committee briefly given above are testimony to the expert knowledge which will be available to deal with problems that must be solved in order that the School of Hygiene may fulfil its purpose in the widest possible way. The members are all men with far-reaching vision, and their recommendations will command public confidence.

Although no doubt the work of post-graduate medical education will be closely connected with the University of London, it is understood that the new school will be governed by a council, which will not be exclusively representative of London University interests, but will also contain men who know Imperial needs and educational necessities, not only in this country, but in all parts of the world.

### THE SUCCESS OF INFANT WELFARE WORK.

NATIONAL BABY WEEK is to be held during the first week of July, and in the next number of this journal we propose to devote special attention to the subject of infant welfare. This subject is of increasing importance in view of the growing number of infant welfare centres in England. The following are the official figures :—

| — | County Council. | Local Sanitary Authority. | Voluntary. | Total. |
|---|---|---|---|---|
| London ... ... | ... | 60 | 155 | 215 |
| County Boroughs | ... | 270 | 157 | 427 |
| Counties ... ... | 349 | 400 | 398 | 1,147 |
| | 349 | 730 | 710 | 1,789 |

The figures for England and Wales since 1916 are as follows :—

| Year. | Municipal Centres. | Voluntary Centres. | Total. |
|---|---|---|---|
| 1916 (Sept.) ... | 396 | 446 | 842 |
| 1917 ... ... | 470 | 480 | 950 |
| 1918 ... ... | 588 | 545 | 1,133 |
| 1919 ... ... | 793 | 619 | 1,412 |
| 1920 ... ... | 1,061 | 693 | 1,754 |
| 1921 ... ... | 1,224 | 736 | 1,960 |

There are signs that last year will prove a record year, with the exception of 1920, for a low infantile death-rate. The death-rates have been: 1917, 104; 1918, 108; 1919, 85; 1920, 75. The provisional figures for 1921 show that the deaths per thousand births for England and Wales were 83, and in London had fallen to 79. But such a low rate can only be maintained by the continued effort of all concerned in work for infant welfare, and it is hoped that the articles to be published next month will be of assistance to all engaged in caring for young children.

The conferences which have been arranged by the National League for Health, Maternity and Child Welfare, to be held at Carnegie House, 117 Piccadilly, W 1, on July 3, 4 and 5, will provide a wealth of information. Mr. Arthur Collins, formerly treasurer to the Birmingham Corporation, is to open a discussion on "The Advisability of Block Grants," a question of considerable importance in view of the recommendations of the Geddes Committee. The problems of rural localities, especially the relation of the district nurse and midwife to the public health services, will be discussed by three of the best-informed medical officers in the country. The debate on "Are Resident Homes for Healthy Babies and Day Nurseries worth the Money they Cost?" will arouse controversy. The need of economy is apparent in the discussion as to the economic value of a successful health visitor, to be opened by Mrs. Eve; in "Child Welfare in Terms of £ s. d." on which Dr. Saleeby and Dr. Edgar L. Collis are to speak; and in the debate with regard to the part taken by voluntary organisations and workers in a complete maternity and child welfare scheme. The programme covers a very wide field, and the speakers are certainly representative of the most expert British opinion.

### THE NATIONAL COUNCIL OF MENTAL HYGIENE.

THE National Council for Mental Hygiene, of which we gave some account in our last issue, has now been inaugurated, with Sir Courtauld Thomson as its first president. At a meeting held in the Barnes Hall of the Royal Society of Medicine, Sir Humphrey Rolleston, President of the Royal College of Physicians, said that the object of medicine at the present time was the prevention of disease, and to that end study was now directed to the detection of very early symptoms, such as functional disorders which preceded any organic change in the body. It was with the detection of early functional disturbances that mental hygiene was closely occupied. It was often said that we were all more or less on the way to being mad. At all events, we must not look on mental disorder as being something rare, or, if it affected ourselves, feel any shame on that account. Since 1908 there had been a Council of Mental Hygiene in America, which had done a great deal of work; four years ago Canada started, and about a year and a half ago a league was founded in France. There were thus three national leagues which were able to meet together and pool their knowledge. The aims of the new Council are:—

(1) The encouragement and the correlation and organisation of means of communication between the various societies and associations concerned with mental hygiene;

(2) To join with the other national councils to form an international league, for combined action and the interchange of knowledge;

(3) To study the causation and prevention of mental disturbances, which were extremely common in this and other countries, and had been increasing since the beginning of the war; including the study of environment, heredity, and various poisons, such as alchohol and lead, the dangerous trades, and the important subject of syphilis;

(4) To include the subject of mental hygiene permanently in medical education;

(5) To further the establishment in general hospitals of special clinics for the early treatment of mental disorders, in such conditions as would remove the public prejudice against the word "mental," which implied that the person was not stable;

(6) To improve the conditions of the treatment of mental disorders, particularly in the early stages, when a great deal of good could be done at home by the institution of social service; and

(7) Judicious propaganda.

A provisional committee, consisting of Sir Norman Moore, Sir Charles Sherrington, President of the Royal Society; Sir George Newman, Principal Medical Officer, Ministry of Health; Sir Walter Fletcher, Secretary of the Medical Research Council; Dr. C. H. Bond, President of the British Medico-Psychological Association; Dr. Bedford Pierce, President of the Section of Psychiatry of the Royal Society of Medicine; Professor George Robertson, President-elect of the British Medico-Psychological Association; Dr. C. S. Myers, Director of the National Institute of Industrial Psychology; Dr. G. Ainsworth, Dr. Helen Boyle, Dr. Edwin Bramwell, Lord Dawson of Penn, Sir Bryan Donkin, Dr. Elliot Smith, Dr. Edwin Goodall, Dr. Henry Head, Dr. Crichton Miller, Dr. W. H. R Rivers, Sir Humphrey Rolleston, President of the Royal College of Physicians; Dr. T. A. Ross, Dr. Tredgold and Dr. W. Worth, was authorised to act for six months, to draw up a constitution and to elect an executive committee.

Communications should be sent to the hon. secretary, National Council for Mental Hygiene, 51 Green Street, London, W.1.

### THE WELFARE OF THE BLIND.

AN important circular was issued recently with regard to the welfare of the blind. The definition of blindness laid down in the Blind Persons Act is "so blind as to be unable to perform any work for which sight is essential." The criteria to be adopted in any particular case are purely objective, having no regard either to the occupation previously followed or to the possibility of the person's being able to follow some special occupation after suitable training or assistance has been afforded to him.

The circular endeavours to lay down certain scales, in order to remedy the present variety in the methods and amounts of payments made in the different workshops for the blind. The Advisory Committee has been seeking to secure more uniformity, and now reaffirms the principle of a sliding scale. It recommends a scale of 15s. a week augmentation of earnings at trade or other standard rates up to 10s. a week, decreasing by 3d. in the shilling to a minimum of 5s. a week where the wages are 50s. a week or more. This scale has been recommended by Sir Alfred Mond to the consideration of local authorities. It is urged that the provision of continuous employment for the blind is of the utmost importance, but inevitably earning capacity differs considerably. The sliding scale insures that fair opportunities are afforded to blind workers to make the best of their capacities.

Fresh arrangements have been made with regard to the registration of blind persons. There is a pleasing absence of red tape in the fact that the Ministry of Health declines to prescribe the exact form of such records and also suggests that in many cases local authorities will find it convenient to delegate the work of compiling and maintaining the records to the local voluntary agencies for the blind which have had considerable experience in this matter. The greatest efficiency can certainly be obtained by municipal care and voluntary benevolence working together.

### THE PROBLEM OF THE TYPHOID "CARRIER."

THERE seems to be little doubt that we must discard the teaching that typhoid fever is usually contracted by contact with water, earth, utensils and the like in which the typhoid bacillus has flourished outside the human body. Typhoid bacilli may, ind ed, live for a short time, but cannot reproduce themselves outside the body. The problem of typhoid prophylaxis is therefore enormously simplified, and if infection by the patient and the "carrier" could be prevented, the disease would soon become practically extinct. In a report, recently issued by Gade's Pathological Institute in Bergen, special attention is paid to the detection and sterilisation of "carriers," the persons, typified by "Typhoid Jane," who trail not "clouds of glory," but disease and death. It has been calculated that only about three per cent. of all patients contracting typhoid fever become chronic "carriers," and by systematic examinations of the excreta there should be little difficulty in distinguishing between the patients who are no longer inf ctious and the potential "Typhoid Janes." Incidentally it may be observed that the overwhelming majority of "carriers" are middle-aged women. Clinical and post-mortem examination of "carriers" have clearly shown that only in a few cases is the infection located to the urinary system, and in these cases urinary antiseptics of he formaldehyde group have often proved effective in sterilising the "carrier." In the vast majority of cases he typhoid bacilli are limited to the gall bladder, and in this position neither vaccines nor antiseptics given by the mouth are of any value. Among 20 cases in which the gall-bladder was moved from "carriers" there were 17 who w e effectively sterilised by this operation. Of the remainder, one died in connection with the operation, and two continued to harbour typhoid bacilli. In view of these operative results, and of the futility of more conservative measures, it is probable that cholecystectomy will in the future occupy an important position in the campaign against typhoid fever, and many a "Typhoid Jane" will prefer this short cut to the prolonged punishment which internm nt inflicts. There are, of course, the risks of the operation; "Typhoid Jane" may prove destitute of altruism, and, invoking these risks, bring *habeas corpus* arguments to bear on the retention of her gall-bladder. Internment as an alternative should be a potent inducement to submit to an operation, and the possibility of an infected gall-bladder provoking disease in the "carrier" herself should further encourage "Jane" to part with her gall-bladder.

In the "Evening News" for May 9 there is a report of a recent judgment promulgated in Illinois by the State Supreme Court. The person concerned, a Mrs. Jennie Barmore, was convicted of being a typhoid "carrier" and condemned to retire to her cottage, to live there alone, and to prepare food for no one but herself. The alternative to voluntary isolation is thus stated in the official decree: "Mrs. Barmore will be locked up permanently if she refuses to stop handling food and does not sterilise her hands. Meanwhile, she can come and go with these restrictions, subject to inspection by health officers." Mrs. Barmore would do well to take medical advice as to the possibility of shedding all her typhoid germs by the removal of her gall-bladder.

---

### THE CAUSATION OF MINERS' BLINDNESS.

A COMMITTEE appointed by the Medical Research Council, consisting of Prof. Haldane, of Oxford (chairman), Prof. Collis, Dr. Llewellyn, Mr. Pooley, F.R.C.S., and Dr. Rivers has published a first statement of its findings. These are unanimous, and are as follows :—

1. The essential factor in the production of miners' nystagmus is deficient illumination. Other factors, such as position during work, accidents, alcoholism, infections, malnutrition, hereditary predisposition, and errors of refraction, are of secondary importance only.

2. The deficient illumination is due to the low illuminating power of the safety lamps generally used by coal-miners, to the distance at which these lamps have to be placed from the objects which the miner has to look at, and to the great absorption of light by the coal. In addition, coal-dust or dirt obscuring the lamp glasses, the choking of the wire gauze chimneys, and moisture or low oxygen percentage in mine air, all reduce the light given by oil lamps, while failing voltage, poor bulbs, or lack of proper attention have similar effects on the illumination given by electric lamps.

3. Workers at the coal face are more affected than other underground workers, and this appears to be due to the unrelieved blackness of the coal and the greater need for accurate vision.

4. Distinct signs of nystagmus are present in a large proportion of coalminers, though only in a small proportion do the symptoms ever become so severe as to cause even temporary incapacity.

The committee recommend that, since incapacity due to nystagmus is rare among coalminers working with open lights, everything possible should be done to make the standard of illumination of the objects looked at equal to that of an open-light pit. This can be effected either by greatly increasing (to about two or three candles) the illuminating power of safety lamps as ordinarily used, or by an electric light fixed on a miner's head, belt, or other convenient position, so that the light is automatically brought nearer the working area and does not impair clear vision by shining directly into the eyes. At parts of the pit other than the coal face the visibility of objects can be greatly increased by whitewashing, as well as by the stone dusting now obligatory for the prevention of explosions. The committee believe that by these means miners' nystagmus of sufficient severity to cause disablement can, by degrees, be entirely prevented.

During their investigations the committee have noticed the prevailing belief among coalminers that miners' nystagmus causes permanent damage to or even total loss of, sight, if underground work is continued after the onset of symptoms. This belief, which is entirely erroneous, has led to much unnecessary suffering and to the development of psychoneurotic symptoms in many cases.

### THE SUPERANNUATION BILL.

THE Local Government and Other Officers Superannuation Bill is now through the Committee stage, and has been reported to the House. It is certainly right that local authorities should have superannuation schemes. Under this Bill, Councils, if they wish, may adopt a scheme which will be uniform for everybody; smaller local authorities may combine, so that there may be a minimum of fifty officers and servants, in order to make it actuarily sound. The officer is to contribute five per cent. on his salary, and the local authority an equivalent amount. Provision is made for reckoning in the back service of existing officers and for payment of the transfer value when an officer transfers from one Council which has adopted the scheme to another.

Such a measure has been demanded by the official associations for many years past. As the measure is only adoptive, it will not mean necessarily extra expense. The Bill is badly drafted, but has improved owing to the fact that the promoters accepted many of the Government amendments.

### THE SCHOOL MEDICAL SERVICES.

"THE Medical Officer" has made a polite reference to a recent comment in this Review in which it was stated that the suggested transfer of the school medical services to the Ministry of Health was quite impracticable. It declares that we are very much out of touch with the public health service. It would be interesting for our contemporary to compare the results of the deputation two months ago to Sir Alfred Mond on this subject with the comments made by us. It would also be valuable if some detailed explanation could be given as to how officials of the Ministry of Health could possibly control without much friction the internal arrangements of schools managed partly by the Board of Education and partly by local education authorities. On questions affecting medical officers we always read our contemporary with care, but on this point there appears to be a lack of appreciation of the practical necessities of administration.

### SAFETY FIRST.

THE annual report of the Registrar-General provides a valuable survey of the health of this country. Those who believe that the value of the study of public health is to preserve and lengthen life must be interested in the table devoted to a comparison between the number of deaths from violence and those from disease. Last year in England and Wales 16,612 persons died from violence as compared with 8,798 from influenza and 1,291 from scarlet fever. In London the rate per thousand of the population of deaths from violence was 0·42 as compared with 0·23 from influenza and 0·06 from scarlet fever. The accident is, in fact, one of the greatest threats to life to-day, and it would be well if more of the public obeyed the warnings of the Safety First Council.

### CHILD WELFARE IN DENMARK.

KING CHRISTIAN IX. recently approved of an Act to regulate the supervision of children boarded out in families that must be of interest to all students of child welfare. All children boarded out have to be under the supervision of the public authorities until they reach the age of 14. The Municipal Council can enlist the services of one or more duly qualified men or women, who have to visit the children frequently, and are allowed free access at any time. Powers are given to the local authority to supervise also any adopted children; but exceptions are allowed in cases where the children have not been taken as a profitable investment. Imprisonment in case of bad treatment can be imposed.

### ALLEGATIONS AGAINST ASYLUM ATTENDANTS.

THE inquiry held by Sir P. J. Willis (chairman), Mr. A. H. Trevor and Dr. Rotheram, two Commissioners of the Board of Control, into the allegations of brutality at the Long Grove Mental Hospital, Epsom, resulted in a decision that the complainant's statements were not corroborated. The attendants denied the charges, and a number of ex-patients and present patients who were in the same part of the building as Mr. Cox (an ex-inspector of police who made the complaints) unanimously stated that they never saw any brutality at all. They also spoke highly of the treatment and kindness they received in the institution. The Court also found that the main charges of cruelty and poisoning of food were untrue. They did not think that Mr. Cox was deliberately stating what he knew to be untrue; nor that he had concocted the statement; but, though he had made the allegations in good faith, they were unable to accept them. There were, however, one or two minor matters in his statement which had some foundation. One was a jesting remark by one of the attendants to a patient whom he asked if he "would like to see the death chamber." The Court thought that such things should not be said even in jest.

It is satisfactory to learn that such serious charges are unfounded. Not that the findings of the Court will satisfy certain critics who are prepared to accept any statements which are made against asylums. Their attitude in the present occasion is that the decision is all wrong; apparently, any verdict given by those connected with the Board of Control could not be right! It is lamentable to have to realise that those who apparently have the welfare of the insane at heart should be so swayed by prejudice. This case should help them to realise the difficulties which have to be faced when accusations of this kind are made and how warily the authorities must proceed. It is a hard enough task to arrive at the truth in an ordinary Court of Law where the witnesses are not swayed by insane prejudice. Even there unsupported testimony is not considered sufficient to bring about conviction. It would be well if the over-enthusiastic reformers were to ascertain if the statements which they are so ready to accept are worthy of credence.

### THE LABOUR PARTY AND THE HOSPITALS.

THE Labour Party has published in the form of a pamphlet a scheme for hospital service with criticisms of the existing system. Those who believe in state control of everything—that is, socialism—will no doubt approve of the views expressed. But those who regard the unregulated activity and enterprise of individuals as the basis of our national prosperity will find much to deprecate. Certainly, of all institutions in the country, hospitals seem the most unsuitable for socialistic experiments, and the pamphlet inadvertently betrays this fact. Thus, after misrepresenting the discipline necessary in the nursing service as servility, the writers state that the needs of the patient outside the medical instructions—which are described as faithfully followed—tend to be ignored, and "a cut and dried unintelligent course" results. Again, the need "for more consideration of the patient as an individual" is proclaimed, and at the same time the demand is made for the state control of hospitals, with its inevitable bureaucratic uniformity. The writers of the pamphlet are evidently ignorant of the fact that this is the one country in Europe where the patient receives great consideration as an individual, while in the state-controlled hospitals of the Continent his individuality tends to be ignored, and he becomes simply "a case." Further, we read of "the evils and odium of the present Poor Law institutions," and of the need for bringing them up to the level of the best voluntary hospitals. It does not seem to have occurred to the writers that the main reason of the superiority of voluntary hospitals is that they are not state-controlled. The paper ends with a grandiose scheme for the organisation of the hospitals under one authority, regardless of such considerations as expense, and the appalling waste of Government management with which we have been only too familiar during and since the war. Curiously, again, as if conscious of the weakness of its programme, the Labour Party hesitates to extinguish completely the voluntary hospitals, for they are given the option of being taken over by the health authorities entirely, of remaining on a voluntary basis entirely, or of receiving grants from public funds conditional on efficiency and the representation of the health authority on boards of management.

### THE BRITISH HOSPITALS ASSOCIATION CONFERENCE.

THE twelfth conference of the British Hospitals Association was held on May 25 and 26 in the banqueting hall of the Exchange Station Hotel, Liverpool, the Hon. Sir Arthur Stanley presiding. The delegates were welcomed by the Lord Mayor of Liverpool, and a paper was read by Sir Alan Garrett Anderson on the need of the hospitals for union, which will be found on page 249 of this issue. In the afternoon, Mr. W. Thelwall Thomas read a paper on "The Voluntary Hospital," and on the second morning there was a paper on "Hospital Economies," by Mr. Frank Hazell Each of the three papers was followed by discussion. On the afternoon of the 26th visits were paid to sixteen local hospitals and infirmaries and to the School of Tropical Medicine (Liverpool University).

Owing to the Conference being held as we go to press, we have to defer publication of the two latter papers and a further report to our next number.

### THE COMBINED APPEAL.

EXCELLENT progress is being made in every branch of this great enterprise. As we go to press, the Empire Flag Day celebrations are in progress Fifty thousand volunteers were asked for to cover the fifteen-mile radius from Charing Cross, and special arrangements were made by the hospitals in order that their students and as many as possible of the nursing staff might take part as flag-sellers. Among coming festivities in aid of the appeal are the Inter-Hospital Sports at Queen's Club on June 10; a ball at the Hyde Park Hotel, to be given by the Queen's Hospital for Children on June 19; and a ball at the Albert Hall on June 28, organised by a committee of which Princess Mary Viscountess Lascelles is president. Messrs. Christies have undertaken to conduct a gift sale at their rooms on a day during the intensive fortnight (June 25–July 8). Gifts, which should be sent to the Combined Appeal headquarters, 19 Berkeley Street, W.1, will be reviewed by a Selection Committee appointed by the King's Fund Council. Articles considered suitable will be handed over to Messrs. Christies, and the remainder will be sold elsewhere for the benefit of the fund. Organisers of entertainments, luncheons, dinners and other functions, who are willing to take collections, are invited to apply to the headquarters for official collection boxes.

The donations already received include an anonymous gift of £5,000.

### THE HOSPITAL PROCESSION.

ON Saturday, July 1, there is to be a Hospital Procession, passing His Majesty at Buckingham Palace. We are glad to see that this has been arranged as one of the chief events during the intensive fortnight of the Combined Appeal for the Hospitals of London, for in our issue of May 7, 1921, we made the suggestion that there should be a Hospital Pageant and Carnival, taking the form of a procession through the principal thoroughfares of London. We proposed that a series of tableaux should be carried on lorries, showing the history of hospitals from the monkish healing of the Middle Ages to the perfected methods of the present day. The suggestion "caught on." Numerous hospital secretaries wrote in approval and agreed that such a pageant would be a valuable part of a scheme of collective publicity on behalf of the Voluntary Hospitals. So much interest was aroused that Mr. Gilbert G. Panter, of the Royal Northern Hospital, convened a meeting of hospital secretaries to discuss the subject. Several meetings followed, and on July 15, at the National Hospital for the Paralysed and Epileptic, a reference committee was elected and charged to form a definite scheme and to ascertain its cost. This committee, which consisted of some of the most active hospital secretaries of the metropolis under Mr. Godfrey Hamilton, as chairman, met many times at "The Hospital" Building, and eventually presented its report to a gathering of hospital representatives at the Mansion House on November 28. The following resolution was carried unanimously :—

"This meeting approves of the recommendations made by the Committee of Hospital Secretaries for the holding of a Pageant and Hospital Week for the benefit of the hospitals of London, subject to the following conditions :—

(*a*) Agreement to support the scheme being received from sufficient Boards of Governors to represent 80 per cent. of the bed accommodation and/or out-patients' attendances of London Voluntary Hospitals.

(*b*) The project being approved by and carried out under the auspices of the King Edward's Hospital Fund for London, which is primarily concerned with the special effort to co-ordinate hospital propaganda."

This resolution was sent to all the Metropolitan hospitals, and on February 8 this year the hon-secretary to the reference committee (Mr. H. E. Smithers) was enabled to forward to the King's Fund the names of no less than 58 hospitals whose committees approved the scheme.

---

### A DISPENSER'S FATAL MISTAKE.

At Westminster, on May 5, an inquiry was held concerning the death of a patient at St. Peter's Hospital, Covent Garden, from the effects of antimony poisoning. Dr. Arthur Herbert Harkness, hon. visiting medical officer at the hospital, said that the man was suffering from bilharzia. The recognised treatment was by intravenous injection of sodium and antimony tartrate in increasing doses. On the last occasion, after several injections had been taken quite well, he flushed up considerably. He was detained in hospital. During the same evening the house surgeon telephoned to Dr. Harkness that certain symptoms has set in. The dispenser, who was very much upset, spoke and said that she had made a miscalculation and had made the solution ten times too strong. The patient died two days later. The dispenser had been with them over a year and had previously given every satisfaction. She had been eight years qualified as a chemist.

The dispenser said it was not until she heard the patient was ill that she began to wonder whether the solution had been correctly made. She denied that she had a bad memory, or that she had been worried about anything. She found she had calculated on the basis of 100 instead of 10.

The Coroner said the dispenser seemed to be competent and had had a great deal of experience. A genuine mistake in no way connected with negligence or recklessness or indifference to consequences was very different from gross and culpable negligence. The jury returned a verdict of "Death from Misadventure."

### SMOKING ROOMS IN NURSES' HOMES.

Mr. Tilbury, Chairman of the Infirmary Committee of the Portsmouth Board of Guardians, was lately perturbed by a woman inspector, who informed the committee that it would have to set apart a smoking room for nurses. Mr. Tilbury is reported to have added that the Guardians did not want to see the nurses smoking, and that only a very few of them desired to do so. The practice of smoking is now general among women, and few Institutions object to smoking by nurses in off-duty hours, if only because such an objection could not be enforced. But evidently Mr. Tilbury was startled, not by the idea of nurses smoking, but by the formality of providing a smoking room for them. We all are ready to wink at practices which it startles us formally to provide for. It would be interesting to know how many nurses' homes attached to voluntary hospitals possess smoking-rooms for their nurses. Such a room is desirable in the interests of those who do not smoke; and now that the practice is generally recognised, it is proper to study the convenience of non-smokers and smokers alike, which can best be done by reserving a room for those who wish to indulge in it. Since smoking entered the hospital wards during the war, it is idle to expect to exclude it from the nurses' homes.

---

### THE REMOVAL OF A BURDEN.

Thanks to Mr. Lyle's amendment, as announced in our last issue, professional nurses will no longer be compulsorily insurable under the Unemployment Insurance Act after July 1. Nurses are congratulating themselves on their release from the annoying obligation to pay for a benefit that, in the nature of their profession, they were highly unlikely to claim. Hospital officials, too, are glad to be relieved of the work connected with the contributions, to say nothing of the expense. Great credit is due to the College of Nursing for its forcible and successful advocacy of the nurses' case for exemption, and we are glad to hear from its energetic secretary, Miss Rundle, that its efforts are widely and gratefully recognised.

---

### IS WATER WASTED IN HOSPITALS?

An extraordinary amount of water, according to a report of the waterworks engineer, is used by King Edward VII. Hospital, Cardiff. The hospital had asked for a concession, and the Waterworks Committee find, on its engineer's authority, that in 1919 the hospital used thirteen million gallons, and that in 1921, this rose to twenty-one millions. The engineer stated that all the other charitable institutions in the city used only eleven millions in 1919, and had used half a million less in the past year. The hospital has been invited to investigate its consumption of water, and to see if a saving could not be made. The Waterworks Committee is opposed to the principle of concessions. The corporation has lately raised its grant to £1,000.

